# Distinct Chemical Changes in Abdominal but Not in Thoracic Aorta upon Atherosclerosis Studied Using Fiber Optic Raman Spectroscopy

**DOI:** 10.3390/ijms21144838

**Published:** 2020-07-08

**Authors:** Krzysztof Czamara, Zuzanna Majka, Magdalena Sternak, Mateusz Koziol, Renata B. Kostogrys, Stefan Chlopicki, Agnieszka Kaczor

**Affiliations:** 1 Jagiellonian Centre for Experimental Therapeutics (JCET), Jagiellonian University, 14 Bobrzynskiego Str., 30-348 Krakow, Poland; zuzanna.majka@student.uj.edu.pl (Z.M.); magdalena.sternak@jcet.eu (M.S.); stefan.chlopicki@jcet.eu (S.C.); 2 Faculty of Chemistry, Jagiellonian University, 2 Gronostajowa Str., 30-387 Krakow, Poland; mateusz.mk.koziol@gmail.com; 3 Department of Human Nutrition and Dietetics, Faculty of Food Technology, University of Agriculture H. Kollataja in Krakow, 122 Balicka Str., 30-149 Krakow, Poland; renata.kostogrys@urk.edu.pl; 4 Chair of Pharmacology, Jagiellonian University, 16 Grzegorzecka Str., 31-531 Krakow, Poland

**Keywords:** perivascular adipose tissue, Raman spectroscopy, fiber optic probe, atherosclerosis, thoracic and abdominal aorta, vascular inflammation

## Abstract

Fiber optic Raman spectroscopy and Raman microscopy were used to investigate alterations in the aorta wall and the surrounding perivascular adipose tissue (PVAT) in the murine model of atherosclerosis (*Apoe^-/-^/Ldlr^-/-^* mice). Both abdominal and thoracic parts of the aorta were studied to account for the heterogenic chemical composition of aorta and its localization-dependent response in progression of atherosclerosis. The average Raman spectra obtained for both parts of aorta cross sections revealed that the chemical composition of intima-media layers along aorta remains relatively homogeneous while the lipid content in the adventitia layer markedly increases with decreasing distance to PVAT. Moreover, our results demonstrate that the increase of the lipid to protein ratio in the aorta wall correlates directly with the increased unsaturation level of lipids in PVAT and these changes occur only in the abdominal, but not in thoracic, aorta. In summary, distinct pathophysiological response in the aortic vascular wall could be uncovered by fiber optic Raman spectroscopy based on simple parameters detecting chemical contents of lipids in PVAT.

## 1. Introduction

Atherosclerosis is a main vascular pathology being an origin, among others, of coronary artery disease and stroke underlying primary causes of death worldwide [1]. Atherosclerosis is a progressive chronic metabolic disorder characterized by accumulation of lipids, inflammation, and endothelial dysfunction leading to fibrosis and plaque formation within arteries [2]. The condition of the cardiovascular system is regulated by the endothelium, the monolayer of highly-specialized cells lining the blood and lymphatic vessels, which triggers atherosclerosis and other lifestyle diseases when dysfunctional [3,4]. In general, all blood vessels in the circulation, except for cerebral arteries and microvessels [5], are surrounded or embedded in perivascular adipose tissue (PVAT). Recent studies have demonstrated that PVAT [6] is also substantially involved in the control of the circulatory system and progression of atherosclerosis [6] suggesting that its occurrence is associated with the disease development [7]. The role of PVAT in atherosclerosis is like a double-edged sword. In healthy organisms under physiological conditions, PVAT exhibits an anti-atherogenic properties by, i.e., the secretion of adiponectin and the contribution to vascular NO production [8,9]. On the other hand, in dysfunctional PVAT, the production of adiponectin is reduced in favor of leptin that is involved in macrophage infiltration and release of pro-inflammatory cytokines [10]. Moreover, the phenotypic and functional differences in PVAT, i.e., brown adipose tissue (BAT)-like characteristic of thoracic aorta (TA) PVAT and white adipose tissue (WAT)-like of abdominal aorta (AA) PVAT, have an impact on susceptibility to atherosclerosis [11]. In pathological conditions—i.e., in obesity—the higher level of fatty acids in arterial circulation leads to excessive accumulation of triacylglycerols in AA PVAT and transformation of TA PVAT into more white phenotype in a process called ‘whitening’ manifested by reduced thermogenesis [12,13].

The key risk factors of atherosclerosis, i.e., obesity [13,14,15,16,17] and hypercholesterolemia (independently to obesity) [18,19,20] result in PVAT inflammation. Due to direct location of PVAT in close vicinity to adventitia, facilitating the flow of adipokines, chemokines, and cytokines [16,21,22,23], inflammation in PVAT undergoes a different mechanism compared to observed in the visceral fat [24] and may easily propagate to the inner layers of the vessel wall triggering dysfunction of the smooth muscle cells and endothelium that give momentum to atherosclerosis development [11]. The apparent influence of PVAT on the vessel wall and direct cross-talk between these tissues [25,26,27] demonstrate that PVAT is a new target of yet unexplored therapeutic potential.

Raman spectroscopy is a label-free and unbiased method that simultaneously measures all components of the sample and provides rich information not only about the sample composition, but also the molecular structure of sample components. It can be used without any sample preparation, for example for analysis of unfixed tissues as PVAT. This makes Raman spectroscopy a unique technique to study tissues; particularly, it is well suited to investigate lipid alterations within. In addition to the detection of lipids, Raman spectroscopy enables to specify the type and structure of lipids—i.e., fatty acids, triacylglycerols, cholesterol and its derivatives, phospholipids, etc. [28]—and can provide quantitative information about the number of double bonds i.e., the degree of lipid unsaturation. It was proven that unsaturation of lipid correlates to the iodine value, an analytical value often used as a measure of unsaturation of lipids [29]. In other work, the lipid unsaturation of lipid droplets obtained from Raman measurements was verified by Oil Red O staining (a ‘gold standard’ method to record the total lipid content) [30]. Moreover, Maslak and colleagues presented an example in which they directly correlated the results obtained from Raman spectra with GC/FID [31]. Hence, spectroscopic techniques have been already validated and present a large potential in terms of possible applications for both: diagnostics as well as routine screening of tissue sections. In our previous works, concentrated on the endothelium, we have demonstrated in various murine models of lifestyle diseases that Raman spectroscopy showed a diagnostic potential via studying effects of chemical changes, i.e., alterations in concentration of biomolecules, induced in tissues under development of studied pathologies, translated into the increase of the specific Raman markers of these diseases [32,33,34,35,36,37,38]. To approach applications of the Raman-based methodology in intraoperative diagnostics, Raman spectroscopy with optical fiber probes has been increasingly used, although at the moment mostly in the context of tumor margin delineation [39,40,41,42]. However, in the proof-of-concept study by Matthäus et al. [43] fiber optic Raman spectroscopy was applied for characterization of atherosclerotic plaque depositions in rabbits in vivo. Moreover, we have also recently used fiber optic probe Raman spectroscopy as an efficient tool for diagnostic of non-alcoholic fatty liver disease, a contraindication in a liver transplant [44]. We have also shown that due to significant Raman cross section for lipids, this methodology is very suitable for studying PVAT [45]. Our results demonstrated that the lipid unsaturation degree was clearly distinct in various types of the adipose tissue (WAT, BAT, TA, AA, and the mesenteric PVAT) and was influenced by the age of animals clearly indicating that aging has a considerable impact on the PVAT’s chemical composition [45]. To prove applicability of Raman-based methodology to study PVAT of internal mammary artery (IMA) or other grafted vessels for purposes of intraoperative diagnostics, fiber optic Raman spectroscopy was used to characterize lipid unsaturation and carotenoid content in PVAT of IMA of patients (*n* = 10) with advanced atherosclerosis and coronary artery disease (Canadian Cardiovascular Society Angina Grading Scale, CCS II or III) undergoing the coronary artery bypass surgery [46]. This study demonstrated that IMA PVAT exhibited varying degrees of the lipid unsaturation and carotenoid content. Most importantly, a high degree of lipid unsaturation and low carotenoid content in PVAT of IMA was found in patients with more advanced coronary artery disease, with CCS class III as compared to CCS class II showing that these markers may reflect the PVAT functional status for patients with advanced coronary atherosclerosis ongoing coronary bypass surgery.

Here, we have used fiber optic Raman spectroscopy to investigate the effects of chemical alterations in the vessel wall and PVAT due to atherosclerosis in mice (*Apoe^-/-^/Ldlr^-/-^* mice). We have confirmed heterogeneity of the chemical composition of murine aorta vessel wall and aortic PVAT and demonstrated resistance of thoracic but not abdominal aorta wall and surrounding PVAT to atherosclerosis-induced changes in lipids profile. Above all, we have proven that pathological changes in lipid content in the vessel wall in abdominal aorta coexist with changes in lipid unsaturation in PVAT. 

## 2. Results and Discussion

### 2.1. Spectral Characteristics of Aorta Layers in Apoe^-/-^/Ldlr^-/-^ Mice 

**Figure 1:**
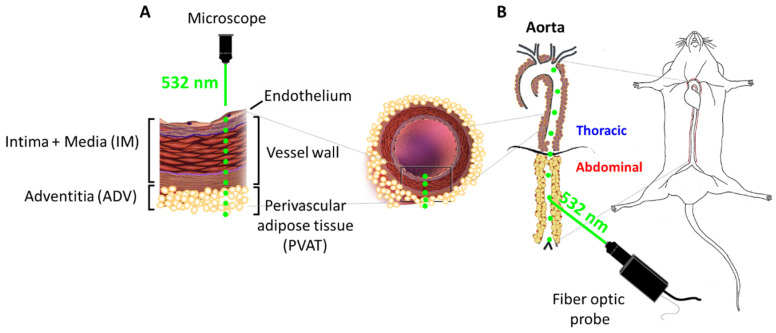
Scheme of the experiment design. An illustrative drawing of the experimental procedure of acquiring Raman spectra using confocal microscope for vessel cross sections (**A**) and Raman fiber optic probe for split-open aorta (**B**). The distance between measured points for the aorta en face was 250 µm, while for the vessel wall and PVAT was 5 and 10 µm, respectively.

To investigate chemical changes in the aorta upon development of atherosclerosis we first performed a comprehensive evaluation of the composition of both thoracic and abdominal regions of the aorta of a control animals. To investigate the composition of the aorta radially from the endothelial layer to PVAT, Raman line mapping of aorta cross sections were done using the confocal Raman microscope (Figure 1A). To analyze the chemical composition along the aorta from the aortic arch to common iliac artery Raman spectra of the split-open aorta wall (the intima/media layer) and surrounding PVAT were collected using the fiber-optic Raman setup (Figure 1B). 

**Figure 2:**
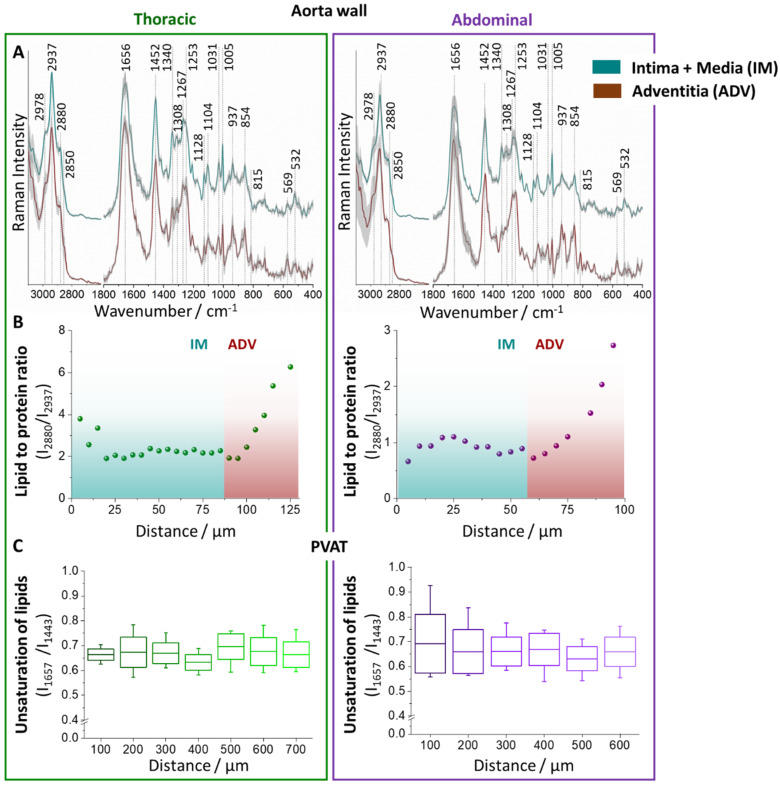
Aorta wall and PVAT chemical characteristics. The average Raman spectra (**A**) and the lipid and protein alterations along the aorta cross section (**B**) in intima and media (IM), and adventitia (ADV) layers of the vessel wall of thoracic and abdominal aorta. The analysis of lipid unsaturation degree of PVAT (**C**). Values given as mean ± SEM are shown in box plots: mean (horizontal line), SEM (box), minimal, and maximal values (whiskers).

Due to their considerably different spectral characteristics, Raman spectra obtained from aorta cross sections were classified as spectra of the PVAT and vessel wall than vessel wall spectra were divided into two subgroups defined as spectra of the ‘inner’ part: intima and media layers (IM), and ‘outer’ part, i.e., adventitia layer (ADV, Figure 2A). In the IM layer, due to considerably smaller thickness of the intima layer, the signal is derived predominantly from the media layer.

Both IM and ADV possess distinct Raman spectral profiles and marker bands, enabling their unambiguous classification that stays in accordance to the microarchitecture of the aorta wall [47]. IM is characterized by higher intensity of the bands at 1128, 1104, and 532 cm^−1^ arising from vibrations of elastin fibers [48], while ADV can be identified by bands at 937, 854, 815, and 569 cm^−1^ originating form collagen [49] with the most distinctive band at 815 cm^−1^ absent in the Raman spectrum of IM. The lipid content in the tissue increases in the (intuitive) order: IM < ADV < PVAT. Assignments of Raman bands of the aorta wall tissue and PVAT are collected in Table S1 (Supplementary Materials).

### 2.2. Distinct Composition of the Adventitia and PVAT in Abdominal and Thoracic Aorta in Apoe^-/-^/Ldlr^-/-^ Mice

The comparison of average Raman spectra obtained for TA and AA revealed that chemical composition of IM along aorta remains relatively homogeneous as the spectra of IM are almost the same and independent on the distance from the endothelium whilst the chemical composition of ADV is considerably different depending on the distance from PVAT. The analysis of lipid to protein ratio (I_2880_/I_2937_) obtained by calculations of the integral intensities of bands at 2880 and 2937 cm^−1^ is presented in Figure 2B. It shows that the lipid content in the ADV markedly increases with the decrease of the distance to PVAT both for TA and AA, demonstrating possible infiltration of PVAT lipids to the adventitia layer. Moreover, the clear discrimination of IM and ADV can be done based on the lipid to protein ratio in the tissue.

The chemical characterization of PVAT was also performed. Due to a substantial number of PVAT Raman spectra obtained from each measurement series, we averaged 10 consecutive spectra (measured with a sampling density of 10 μm) and used the averaged spectra for the calculations of the intensity of bands at 1657 and 1443 cm^−1^ to determine lipid unsaturation (Figure 2C). The lipid to protein marker in this case is not valid as the protein content in PVAT is very low. In general, Figure 2C shows that the chemical composition of PVAT is quite uniform and homogenous in the studied scale.

This important part of the study demonstrates that IM and PVAT, but not ADV, are chemically rather homogenous. Therefore, IM and PVAT were investigated using the fiber optic Raman spectroscopy to analyze the impact of atherosclerosis on local changes in the aorta in the abdominal and thoracic parts.

### 2.3. Media Layer in Abdominal, but not Thoracic, Aorta Display Alterations in Lipid Content in Apoe^-/-^/Ldlr^-/-^ Mice 

**Figure 3:**
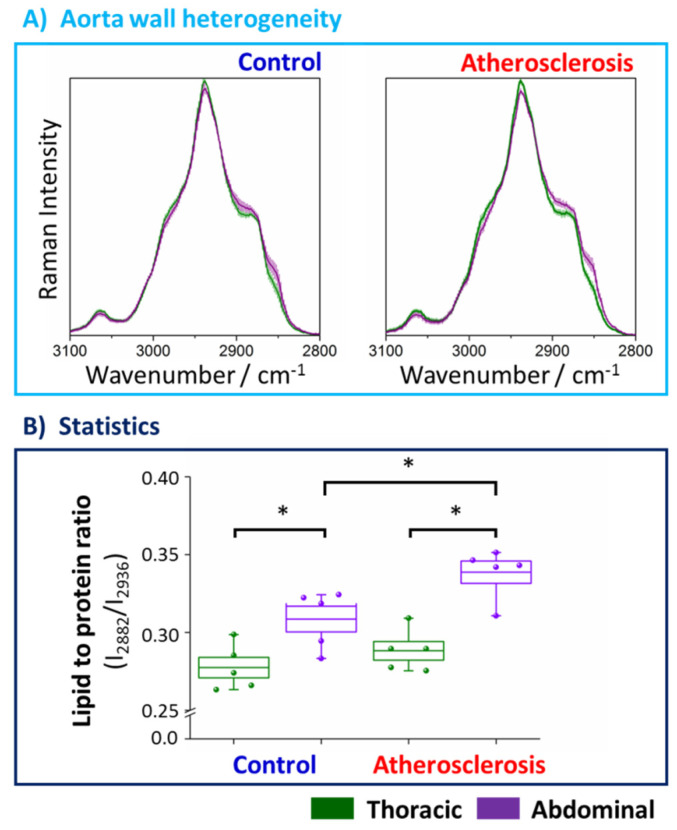
Averaged Raman spectra and the statistical analysis of the lipid to protein ratio of the thoracic and abdominal fragments of the aorta wall upon atherosclerosis development. Averaged Raman spectra of the thoracic (green) and abdominal (violet) aorta wall obtained from the control group (blue) and animals with developed atherosclerosis (red) (**A**). Spectra were normalized and presented with the standard deviation on each data point (accordingly lighter color). The analysis of the lipid to protein ratio (**B**) was calculated using the integral intensities of bands at positions 2882 and 2936 cm^−1^. Values were shown in the box plots: mean (horizontal line), SEM (box), minimal and maximal values (whiskers). The level of statistical significance: * *p* <0.05.

To define the chemical changes due to the progression of the atherosclerosis, fiber optic Raman spectroscopy was applied for point by point measurement of the vessel wall along the long axis from the endothelial side (the en face aorta). The Raman spectra in the high-wavenumber range averaged over 5 animals (24 spectra per animal) of the thoracic (denoted green) and abdominal (denoted violet) parts of the aorta wall are presented in Figure 3 (Figure S1, Supplementary Materials, shows also the fingerprint range). 

Values of integral intensities of bands at 2882 and 2936 cm^−1^, assigned mostly to lipids and proteins, respectively, were used in the statistical analysis as the markers of the chemical composition of the tissue. Standard deviations of the spectra between animals from the same group are relatively small which reflect small spectral intravariability per group. However, differences between spectra per animal are relatively high (Figure S2, Supplementary Materials), therefore we averaged several (24) spectra per animal. Figure 3A shows the comparison of Raman signatures of the averaged thoracic and abdominal fragments of the aorta wall indicating clearly that the chemical composition of the thoracic and abdominal aorta both for the control group and, particularly for the mice with developed atherosclerosis, is different.

The influence of the atherosclerosis on the chemical composition of thoracic and abdominal parts of the aorta wall was studied. Clearly, there are no considerable alterations in the Raman signatures and, therefore, in the composition of the TA, whilst in the AA subtle changes in the averaged Raman spectrum are observed (Figure S2A, Supplementary Materials). To explore semi-quantitatively this effect, integral intensities of the components of the bands at positions 2882 cm^−1^ (mostly due to lipids) and 2936 cm^−1^ (mostly due to proteins) were calculated and presented as the ratio in Figure 3B. The lipid to protein ratio is significantly bigger for the abdominal aorta compared to the thoracic part demonstrating clearly heterogeneity of the aorta composition and showing that the wall of the abdominal aorta contains more lipids compared to the thoracic aorta (Figure 3A) and this effect is more accentuated for subjects with developed atherosclerosis (17.7%) relatively to the control group (11.6%). Moreover, the lipid to protein ratio significantly changes (9.7%) in the abdominal (but not thoracic) part of the aorta wall showing that progression of the disease affects the AA, whilst the TA is resistant to atherosclerosis-induced changes in lipid content, even at the late stage of the atherosclerosis development (22 weeks). The increased lipid content in the abdominal part is also reflected in the fingerprint spectral region where the signal at 704 cm^−1^ arising from cholesterols is clearly visible for the atherosclerotic group (Figure S1B, Supplementary Materials). Therefore, the accumulation of lipids (among others, cholesterol) occurs in the abdominal part of the aorta, but this effect is not observed for the thoracic part that seems to be resistant for lipid accumulation even at this late level of atherosclerosis progression as evidenced by fiber optic Raman spectroscopy. 

It is known that atherosclerosis develops faster in the abdominal aorta compared to the thoracic one [50], which is consistent with the biochemical studies on endothelial function in this model indicating more pronounced endothelial dysfunction in the abdominal aorta in *Apoe^-/-^/Ldlr^-/-^* mice [51]. To answer the question of how these changes in the aorta wall correlate with alterations in the perivascular adipose tissue, PVAT from the thoracic and abdominal fragments was extracted and investigated. 

### 2.4. Different PVAT Chemical Composition in Abdominal and Thoracic Aorta in Apoe^-/-^/Ldlr^-/-^ Mice

The response of the various adipose tissue depot to the development of atherosclerosis is not uniform. In particular, BAT and WAT seem to play opposite roles, anti- and pro-atherosclerotic, respectively [11]. Obesity results in hypertrophy and increased lipolysis of WAT adipocytes as well as their reduced response to insulin [11]. Overall, WAT adipocytes contribute to development of atherosclerosis by elevation of a free fatty acids level in plasma. Contrarily, BAT-mediated non-shivering thermogenesis consumes free fatty acids and glucose decreasing their plasma level and reversing the WAT action [19,20]. The antiatherogenic effect can be obtained by stimulating BAT and conversion of the adipose tissue phenotype toward the brown one (‘beiging’). Importantly, PVAT also possesses a considerably different and convertible phenotype depending on the localization and age [45,52] and, additionally, is in a direct contact with the vessel wall, which may enhance its paracrine effect [11].

**Figure 4:**
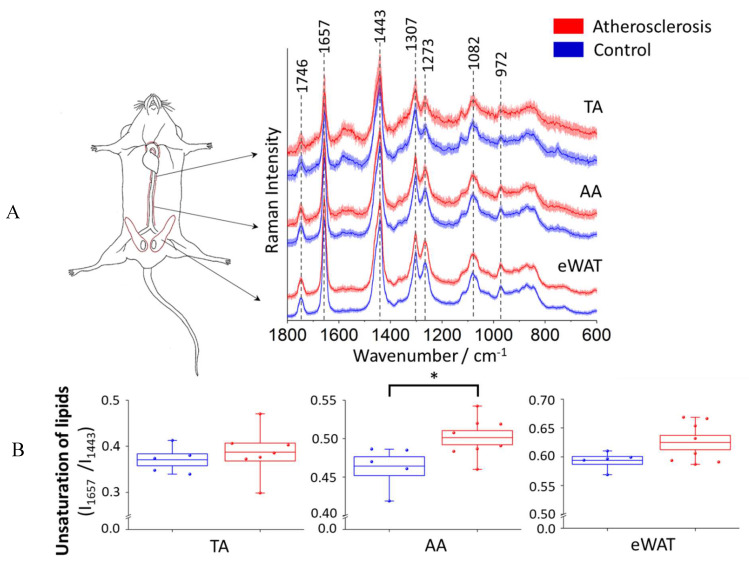
Averaged Raman spectra of the thoracic and abdominal PVAT and epididymal adipose tissue and statistical analysis of unsaturation of lipids. Averaged Raman spectra (**A**) in the fingerprint region of the thoracic (TA) and abdominal (AA) PVAT and the epididymal adipose tissue (eWAT) for the control group (blue) and animal with developed atherosclerosis (red) presented with the standard deviation on each data point. The analysis of the lipid unsaturation level (**B**) calculated as the ratio of integral intensities of bands at 1657 and 1443 cm^−1^. Values were shown in box plots: mean (horizontal line), SEM (box), minimal and maximal values (whiskers). The level of statistical significance: * *p* < 0.05.

In order to investigate the impact of atherosclerosis on perivascular adipose tissue, in particular TA and AA parts of PVAT, the averaged Raman spectra (5 animals in each group, at least 7 spectra per animal) of TA and AA PVAT were analyzed (Figure 4). Additionally, the white adipose tissue surrounding epididymis (eWAT) was studied for comparison.

The collected Raman spectra of PVAT exhibit homogeneity and an increased signal to noise ratio observed for the spectrum of the thoracic aorta may be due to a quite high content of hemoproteins in its mitochondria-rich ‘BAT-alike’ structure. The presence of hemoproteins is revealed by characteristic bands at 1585, 1131, and 751 cm^−1^ and the increased fluorescence background (532 nm excitation was used) [53,54]. As it was previously reported [45], the averaged Raman spectra of the adipose tissue reflect the typical profile of unsaturated triacylglycerols [28] that are main components in the adipose tissue (Figure 4A). Additionally, in line with our previous study [45], the unsaturation level of the adipose tissue for the control group follows the order eWAT > AA > TA. A semi-quantitative estimation of the ratio of 1657 and 1443 cm^−1^ bands, reflecting the lipid unsaturation degree [28,45], is shown in Figure 4B. The results exhibit that the change of the lipid unsaturation ratio is the most prominent (and statistically significant) for the abdominal PVAT (8.0%). For eWAT, a slight (5.2% and statistically insignificant) increase in the lipid unsaturation is observed, whilst for the thoracic PVAT there is practically no change in this parameter. Therefore, although the basal level of lipid unsaturation is lower in the abdominal PVAT than in eWAT, the phenotype of the abdominal PVAT (reflected in this case in lipid unsaturation) converts readily into WAT-like phenotype, i.e., considerable whitening of the abdominal (but not thoracic) PVAT occurs due to atherosclerosis progression. A direct comparison between atherosclerosis-induced alterations in the inner layers (intima and media) and surrounding PVAT is made below.

### 2.5. Alterations in PVAT and Vessel Wall Coexist in Abdominal Aorta in Apoe^-/-^/Ldlr^-/-^ Mice

**Figure 5:**
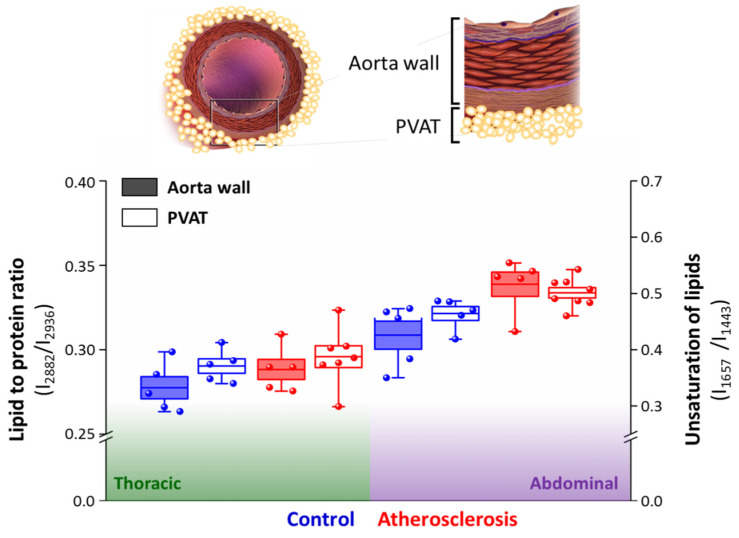
Correlation of atherosclerosis-induced alterations in the aorta wall and surrounding PVAT. The comparison of the lipid to protein ratio of the aorta wall tissue and unsaturation of lipids of PVAT were calculated using the ratio of integral intensities of bands at 2882–2936 cm^−1^ and 1657–1443 cm^−1^, respectively. Values were shown in box plots: mean (horizontal line), SEM (box), minimal and maximal values (whiskers). Significance of pairwise analysis are presented in Figure 3 and Figure 4.

Overall, the impact of atherosclerosis both on the aorta wall and surrounding PVAT is significantly manifested only in the abdominal part of the aorta (Figure 5). Contrarily, the thoracic part of the aorta and PVAT is resistant to atherosclerosis-induced changes in lipid content, as shown by lack of chemical changes in the Raman spectra of these tissues. WAT-like (i.e., more prone to inflammation, antiatherogenic) and BAT-like (i.e., resistant to inflammation, proatherogenic) phenotypes of the adipose and thoracic parts of PVAT [11], respectively, are reflected in the different basal levels of lipid unsaturation in these tissues. The increase of the unsaturated lipid content is associated with increased lipid acid desaturase activity and indicates inflammation as shown previously studying non-alcoholic fatty liver disease progression [55]. Analogically, the augmented level of the lipid unsaturation ratio is a hallmark of inflammation in endothelial cells [56].

Strikingly, the increase of the unsaturation level of lipids in PVAT correlates directly with the increase of the lipid-to-protein ratio in the aorta wall, comparing both the basal levels and changes in the respective values (Figure 5). It was demonstrated previously on big cohorts of patients with atherosclerosis and diabetes that although adiponectin serum levels in these pathologies are inversely correlated with the vascular NADHP oxidase activity and superoxide ^•^O_2_^−^ levels, PVAT responses to the increased NADHP oxidase activity in underlying vessels via upregulation of adiponectin gene expression [25,27]. Vascular upregulation of PVAT adiponectin clearly demonstrates the existence of a direct cross-talk between the vascular arterial wall and the surrounding PVAT [25,27].

Apparently, these interactions between the vessel wall and the surrounding PVAT [25,27] are reflected also in considerable and consistent changes in the chemical composition of the intima/media layer and PVAT, which may be important in the context of future diagnostic and therapeutic strategies.

## 3. Materials and Methods 

### 3.1. Preparation of Samples

The samples of the aorta with perivascular adipose tissue (PVAT) and epididymal white adipose tissue (eWAT) were isolated from male mice with developed atherosclerosis (murine model *Apoe^-/-^/Ldlr^-/-^*) at the age of 22 weeks (*n* = 5). Male C57Bl/6 age matched animals (*n* = 5) were taken as a control. In order to exclude additional factors that could disturb the results, and because the male mice model is more stable, we used only males. The *Apoe^-/-^/Ldlr^-/-^* murine model developed initially by Ishibashi and coworkers [57] represents a validated model of murine atherosclerosis as shown in numerous previous studies from our group using various methodologies [51,58,59,60] including also Raman and IR spectroscopy [32,61,62].

After the resection, the aorta was rinsed from the blood residue inside the vessel and cleansed from the surrounding adipose tissue. Then, the aorta was split open along the axis of blood (the aorta en face patches). The expanded blood vessel was placed on a Cell-Tak^®^-coated CaF_2_ slide so that the endothelial layer faced up. Placed on the plate, the tissue fragment was rinsed with physiological saline and then fixed with a 4% formaldehyde solution for 10 min. The final step was to rinse the tissues twice with distilled water and leave to dry. PVAT from the thoracic part of the aorta and the abdominal cavity were identified as the thoracic and abdominal PVAT, respectively [63]. The fresh fragments of the PVAT adipose tissue were rinsed in NaCl isotonic solution and placed on a CaF_2_ slide. The same procedure as for PVAT was applied to the epididymal white adipose tissue (eWAT) which was taken from depots surrounding epididymis and testicles [63]. Such prepared (unfixed) fragments were measured by fiber optic Raman spectroscopy. All experimental procedures involving animals were conducted according to the Guidelines for Animal Care and Treatment of the European Communities and the Guide for the Care and Use of Laboratory Animals published by the US National Institutes of Health (NIH Publication No. 85–23, revised 1996). All procedures were approved by the Local Ethical Committee on Animal Experiments (permit 212/2105).

To establish the chemical alterations in the aorta wall and surrounding perivascular adipose tissue freshly isolated aortas with PVAT from control C57BL/6 mice (*n* = 3) were studied. Fragments of the thoracic and abdominal aorta were rinsed with PBS, cut into ca. 2 mm cross section slices, placed onto Cell-Tak^®^-coated CaF_2_ slides, and maintained in PBS until Raman measurements.

### 3.2. Instrumentation

Samples of the aorta wall and PVAT were measured using a prototype of a portable WITec Alpha Cart system. The system is equipped with a low-noise CCD detector (Andor, Oxfordshire, England), spectrograph (600 lines per mm grating), air-cooled solid state laser with an excitation wavelength of 532 nm. Laser light was provided via a photonic fiber (3.5 μm) and focused onto a sample with a fiber optic Raman probe tipped with an air objective (Zeiss, 10× magnification, NA = 0.23). Spectra were obtained by averaging 30 accumulations with 1 s integration time, using the maximum laser power of ca. 28 mW. For PVAT at least seven good quality spectra for each animal were taken for analysis. Raman spectra of the aorta wall were recorded along the tissue, i.e., from the thoracic section to the abdominal section with the step of 250 µm. The first 24 spectra were identified as the thoracic section and last 24 as the abdominal section. The 24 spectra of each section were averaged.

The Raman measurements of fresh cross sections of the aorta wall with PVAT were performed with confocal Raman system WITec Alpha300 equipped with a CCD detector (DU 401-BV, Andor, Oxfordshire, England), a 600 mm^−1^ grating and a 63× water immersive objective (Zeiss, NA = 1.0). Spectra were collected point-by-point from the artery lumen to the edge of PVAT every 5 and 10 μm for aorta wall and PVAT, respectively. Samples were illuminated with air-cooled solid-state laser with an excitation of 532 nm using ca. 28 mW power. For acquisition of each Raman spectra 10 accumulations and 1 s integration time were used.

### 3.3. Data Analysis

Preprocessing was done using the WITec Project Plus software and included baseline-correction using autopolynomial of degree 3 and cosmic ray removal procedure. In the second stage, Raman spectra were normalized using vector normalization in the 3100–2800 cm^−1^ or 1800–400 cm^−1^ spectral ranges using the OPUS 7.2 program (Billerica, MA, USA). Furthermore, in the averaged spectra of various tissue fragments, the integral intensity of the bands in the ranges of 3030–2900 cm^−1^, 2900–2840 cm^−1^; and at 1660, 1445, and 1519 cm^−1^ were calculated. The following ratios of bands: 2900–2840/3030–2900 cm^−1^ and 1660/1445 cm^−1^ were used to determine the lipid to protein ratio and unsaturation of lipids, respectively. The data were compared in the Origin Pro 9.1 program (Northampton, ma, USA) using the ANOVA variance analysis with the Tukey post hoc test. If the *p* parameter was at most 0.05, differences were identified as statistically significant.

## 4. Conclusions

Susceptibility to atherosclerosis of the abdominal aorta was previously demonstrated using other methods—i.e., MRI, AFM, and EPR—presenting multifactorial response involving inter alia endothelial stiffness and diminished glycocalyx coverage at the early stage of disease before the development of atherosclerotic plaques [51]. Our work taking advantage of fiber-optic Raman spectroscopy underlines that the abdominal and thoracic parts of the aorta respond differently for the atherosclerosis-induced changes in lipid content. Although in control mice the thoracic part of the vessel wall (both IM and ADV) has a higher basal lipid content than the abdominal part, in studied *Apoe^-/-^/Ldlr^-/-^* mice with atherosclerosis only the abdominal aorta displays changes in lipids that were surprisingly present in PVAT (the increase of unsaturation ratio) as well as in the vascular wall (the increase in the lipid-to-protein ratio). These results underscore a possible primary role of PVAT in the alterations of the lipid contents of the vessel wall. Accordingly, fiber optic Raman spectroscopy based on simple parameters detecting chemical contents of lipids in PVAT may provide a quick test to exclude or suggest a possible alterations in lipid composition in the media layer of the vascular wall. This hypothesis will be further studied in a clinically-relevant context.

## Supplementary Materials

Supplementary materials can be found at https://www.mdpi.com/1422-0067/21/14/4838/s1.

## Abbreviations

AA	Abdominal aorta
ADV	Adventitia layer of aorta wall
*Apoe^-/-^/Ldlr^-/^*	ApoE and LDL receptor double knockout mice
BAT	Brown adipose tissue
CCS II/III	Canadian Cardiovascular Society Angina Grading Scale II or III
eWAT	Epididymal white adipose tissue
IM	Intima and media layers of aorta wall
IMA	Internal mammary artery
NO	Nitric oxide
PVAT	Perivascular adipose tissue
TA	Thoracic aorta
WAT	White adipose tissue
