# Supplementary material for: Distinct Chemical Changes in Abdominal but Not in Thoracic Aorta upon Atherosclerosis Studied Using Fiber Optic Raman Spectroscopy
Source: Int J Mol Sci. 2020 Jul 8;21(14):4838. doi: 10.3390/ijms21144838 (PMC7402309; doi:10.3390/ijms21144838)
Supplement: Supplementary file 1 [file ijms-21-04838-s001.pdf]

Supplementary Materials

Distinct chemical changes in abdominal but not in thoracic aorta upon atherosclerosis studied using fiber optic Raman spectroscopy

Krzysztof Czamara[1,#,*], Zuzanna Majka[1,#], Magdalena Sternak[1], Mateusz Koziol[2], Renata Kostogrys[3], Stefan Chlopicki[1,4] and Agnieszka Kaczor[1,2,*]

[1]Jagiellonian Centre for Experimental Therapeutics (JCET), Jagiellonian University, 14 Bobrzynskiego Str., 30-348 Krakow, Poland.

[2]Faculty of Chemistry, Jagiellonian University, 2 Gronostajowa Str., 30-387 Krakow, Poland.

[3]Department of Human Nutrition, Faculty of Food Technology, University of Agriculture H. Kollataja in Krakow, Balicka 122, Krakow, 30-149, Poland.

[4]Chair of Pharmacology, Jagiellonian University, 16 Grzegorzecka Str., 31-531 Krakow, Poland.

[#] Authors contributed equally.

* correspondence: agnieszka.kaczor@uj.edu.pl, krzysztof.czamara@uj.edu.pl

Table S1. Assignment of Raman bands observed for aorta wall and PVAT tissues.

Wavenumber / cm^{-1}	Vibration type	Assignment
2978	$\nu_{as}(CH_3)$	Proteins, lipids
2937	$\nu_s(CH_3)$	Proteins, lipids
2880	$\nu_{as}(CH_2)$	Proteins, lipids
2850	$\nu_s CH_2$	Proteins, lipids
1746	ν(C=O)	Lipids (triacylglycerols)
1656/1657	Amide I; ν(C=C)	Proteins; unsaturated lipids; water
1452/1443	$\delta(CH_2/CH_3)$	Proteins, lipids
1340	$\delta(C_\alpha$-H); A, G	Proteins; nucleic acids
1308/1307	$\tau(CH_2)$	Lipids
1273	δ(=C-H)	Unsaturated lipids
1267	Amide III	Proteins
1253	Amide III	Proteins
1128	ν(Cα-H)	Elastin (isodesmosin, desmosine)
1104	ν(Cα-H)	Elastin (isodesmosin, desmosine)
1082	ν(C-C)	Lipids
1031	δp phenylalanine; ν(C-N)	Proteins; nucleic acids
1005	δp phenylalanine	Proteins
972	β(C-H)	Lipids
937	ν(C-C) backbone	Proteins (collagen)
854	δp tyrosine	Proteins
815	ν(C-C) backbone	Proteins (collagen)
569	ν(C-C) backbone	Proteins (collagen)
532	ν(S-S)	Elastin

ν - stretching, δ – deformation (δp – ring deformation), β – bending; A - adenine, G – guanine

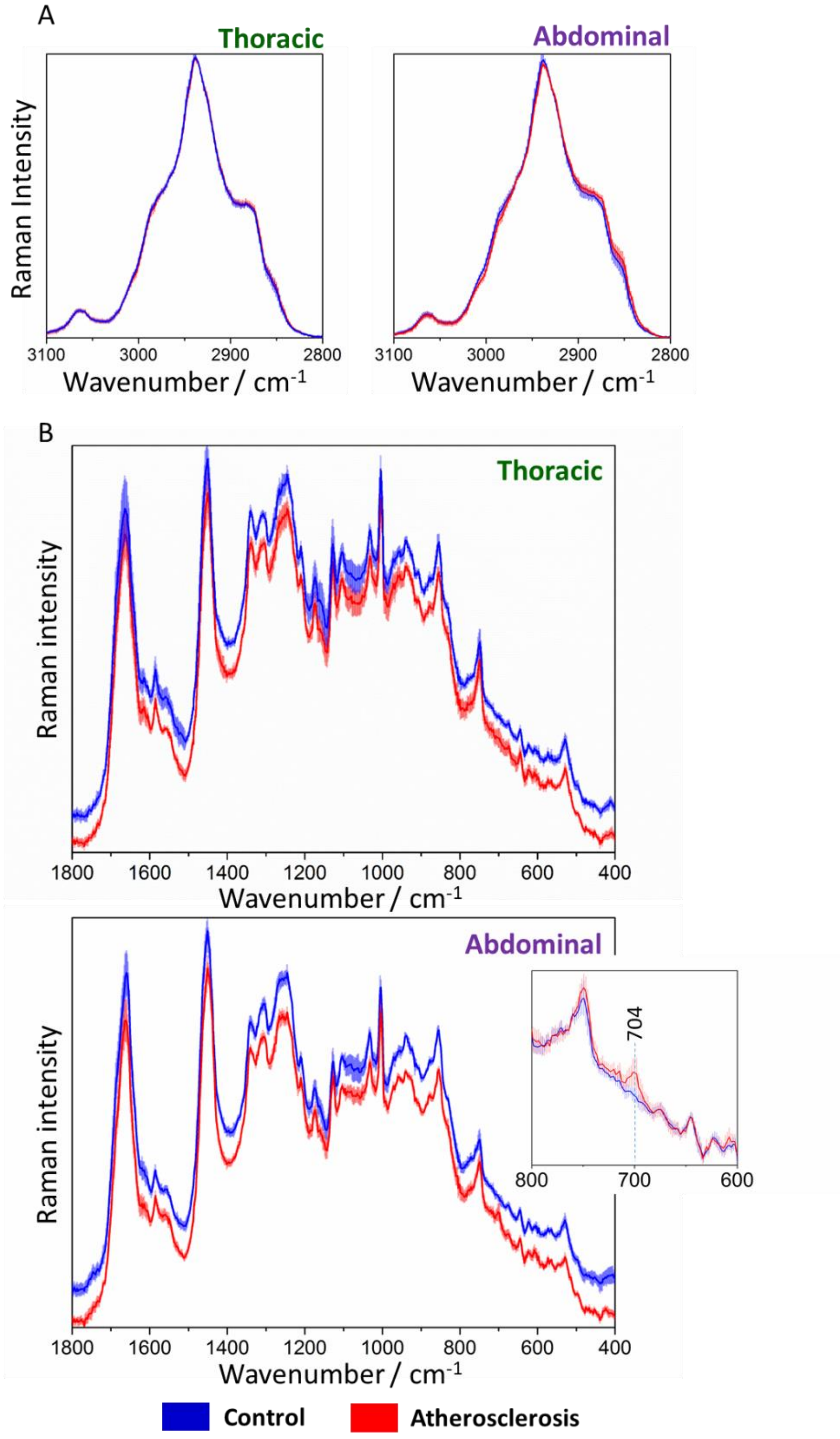

Fig. S1. Raman spectra of the thoracic and abdominal aorta wall. Averaged Raman spectra of the high wavenumber spectral region (A) and the fingerprint spectral range (B) of the thoracic (green) and abdominal (violet) parts of the aorta wall obtained from the control group (blue) and animals with developed atherosclerosis (red)**.** Spectra were normalized and presented with the standard deviation on each data point (accordingly lighter color).

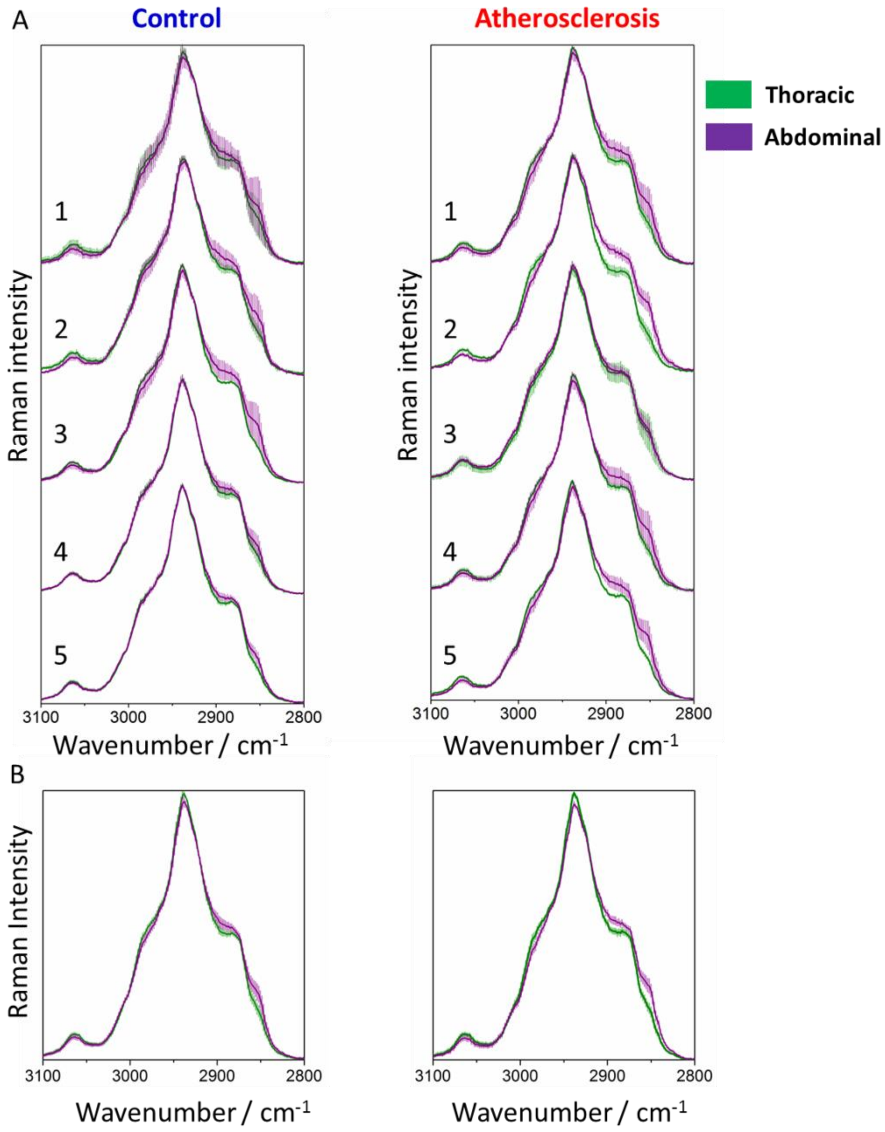

Fig. S2. Variability of the thoracic and abdominal aorta wall spectral profile within the sample from one mice. The comparison of the averaged spectra in the high-wavenumber spectral region of all individual mice (A) and averaged spectra (B) of the thoracic and abdominal part aorta from control and atherosclerosis group with standard deviation on each data point of the averaged spectra.
